# *ERCC1* rs11615 polymorphism increases susceptibility to breast cancer: a meta-analysis of 4547 individuals

**DOI:** 10.1042/BSR20180440

**Published:** 2018-06-21

**Authors:** Bingjie Li, Xiaoqing Shi, Yingying Yuan, Mengle Peng, Huifang Jin, Dongchun Qin

**Affiliations:** 1 Department of Clinical Laboratory, The First Affiliated Hospital of Zhengzhou University, Key Laboratory of Laboratory Medicine of Henan Province, Zhengzhou, Henan, China; 2 Department of Clinical Laboratory, The Third People’s Hospital of Henan Province, Zhengzhou, Henan, China; 3 Department of Blood Transfusion, The First Affiliated Hospital of Zhengzhou University, Zhengzhou, Henan, China

**Keywords:** Breast cancer, ERCC1, Meta-analysis, Polymorphism, Susceptibility

## Abstract

Excision repair cross-complementation group 1 (ERCC1), a DNA repair protein, is vital for maintaining genomic fidelity and integrity. Despite the fact that a mounting body of case–control studies has concentrated on investigating the association of the *ERCC1* rs11615 polymorphism and breast cancer risk, there is still no consensus on it. We conducted the current meta-analysis of all eligible articles to reach a much more explicit conclusion on this ambiguous association. A total of seven studies involving 2354 breast cancer cases and 2193 controls were elaborately selected for this analysis from the Embase, EBSCO, PubMed, WanFang, and China National Knowledge Infrastructure (CNKI) databases. Pooled odds ratios (ORs) and their 95% confidence intervals (CIs) were estimated in our meta-analysis. We found that the *ERCC1* rs11615 polymorphism was significantly associated with breast cancer risk under all genetic models. When excluded, the studies that deviated from Hardy–Weinberg equilibrium (HWE), the pooled results of what remained significantly increase the risk of breast cancer under the allele model (OR = 1.14, 95% CI = 1.02–1.27, *P*=0.02), heterozygote model (OR = 1.24, 95% CI = 1.06–1.44, *P*=0.007), and dominant model (OR = 1.21, 95% CI = 1.05–1.41, *P*=0.01). This increased breast cancer risk was found in Asian population as well as under the heterozygote model (OR = 1.24, 95% CI = 1.05–1.48, *P*=0.013) and dominant model (OR = 1.20, 95% CI = 1.02–1.42, *P*=0.03). Our results suggest that the *ERCC1* rs11615 polymorphism is associated with breast cancer susceptibility, and in particular, this increased risk of breast cancer existence in Asian population.

## Introduction

Breast cancer is one of the most common malignancies in females and it alone accounts for 25% of all the cancer cases as well as 15% of cancer deaths amongst females [1,2]. Although early detection through mammography and improved treatment have contributed to the decrease in breast cancer death rate in Europe [3,4], we are still confronted with a high incidence of new breast cancer diagnoses, causing it to be a major public health problem. Numerous studies have shown that the genetic, endocrine, and external environments contribute to the occurrence and development of breast cancer [5,6]; however, the specific mechanisms amongst these multiple factors are still a mystery. Genomic instability is a potential carcinogenic factor; organisms have developed an elaborated set of DNA repair systems involving multiple sophisticated mechanisms for repairing an extremely broad array of DNA lesions induced by internal and external stressors to maintain genome integrity and stability [7,8]. A series of proteins assemble and respond to DNA damage in a stepwise fashion in these pathways, and the alteration of genes encoding such proteins undoubtedly contributes to the variability of the more directly implicated genes and may therefore be significantly related to the risk of cancer [9].

Excision repair cross-complementation group 1 (ERCC1) is a critical DNA repair protein and is involved in several distinct DNA-damage repair pathways, including nucleotide excision repair (NER), base excision repair (BER), interstrand cross-link (ICL) repair, and recombinational DNA repair [10–13], in the form of a highly conserved heterodimeric complex that combines with the xeroderma pigmentosum complementation group F (XPF) endonuclease. The structure-specific endonuclease ERCC1-XPF serves as an indispensable component in a given DNA repair pathway by catalyzing the incision of the 5′-phosphodiester backbone around the site of DNA lesions caused by a variety of environmental carcinogens and chemotherapeutic agents [14,15]. Previous research revealed that *ERCC1* gene polymorphisms were associated with reduced mRNA and protein expression levels in various types of carcinomatosis [16–18]. The immediate impairment in DNA repair capacity on account of genetic variation may contribute to interindividual variability in cancer susceptibility.

A number of epidemiological studies and meta-analyses of the association between *ERCC1* gene polymorphisms and the risks of several types of cancer such as lung cancer, adult glioma, colorectal cancers, bladder cancer, and head and neck carcinomas have been reported [19–24]. In addition, a pooled result showed that within Caucasian population, individuals with the *ERCC1* rs3212986 gene polymorphism suffer a higher risk of breast cancer [25]. Several case–control studies focussed on the *ERCC1* rs11615 gene polymorphism and breast cancer risk have produced mutually contradictory results [26–32]. Thus, we performed this meta-analysis based on the available case–control studies to expound the effect of the *ERCC1* rs11615 polymorphism on breast cancer risk.

## Materials and methods

### Search strategy

We searched for relevant articles published in the Embase, EBSCO, PubMed, WanFang, and China National Knowledge Infrastructure (CNKI) databases up to December 2017, using the following terms: ‘breast carcinoma’ or ‘breast cancer’ or ‘breast neoplasm’ AND ‘polymorphism’ or ‘genetic variant’ or ‘single-nucleotide polymorphism’ (‘SNP’) AND ‘*ERCC1*’ or ‘354 T>C’ or ‘G19007A’ or ‘rs11615’. All the relevant studies were retrieved for screening of the abstracts, full-text reports, and references by two investigators independently. Authors were contacted to obtain relevant data not present in the original articles. There was no language restriction on the search and selection of the articles.

### Selection criteria

An article was included in our meta-analysis if it met the following selection criteria: (i) the study evaluated the association between the *ERCC1* rs11615 polymorphism and breast cancer risk; (ii) the study was an independent case–control study for humans; and (iii) genotype frequencies of case and control groups were stated in the article or could be obtained by contacting the authors. We excluded reviews, conference papers, and other studies that were published in abstracts only. When publications had obvious overlapping data in terms of the study participants, we kept only the study with the largest sample size.

### Data extraction

A standardized form was used for information collection from each retrieved study by two investigators independently. We collected information on the first author’s name, year of publication, ethnicity (Caucasian, Asian, and others), country, total number of cases and controls, genotyping method, the distribution of genotypes in cases and control subjects, evidence of Hardy–Weinberg equilibrium (HWE) in the control group, and association with breast cancer. During the data extraction, there was an open discussion amongst all the investigators for reaching a final consensus regarding discrepancies.

### Statistical analysis

All statistical analyses were performed using STATA version 12.0 software (StataCorp LP, College Station, TX). Pooled odds ratios (ORs) with their 95% confidence intervals (CIs) were used to estimate the association between the *ERCC1* rs11615 polymorphism and breast cancer risk in the allele model (T compared with C), homozygous model (TT compared with CC), heterozygous model (TC compared with CC), recessive model (TT compared with TC + CC), and dominant model (TC + TT compared with CC); *P*<0.05 was considered significant. We assessed HWE in control subjects of each study by the χ^2^ test (https://ihg.gsf.de/cgi-bin/hw/hwa2.pl; accessed 10 January 2018) [28] and evaluated whether the results were different after excluding studies that had statistically significant (*P*<0.05) violation of HWE [33]. The statistical heterogeneity amongst studies was evaluated with the *Q* statistic based on a standard Chi-square test with a *P*<0.10 [34] and *I^2^* values that manifested the definite extent of between-study heterogeneity [35]. A random-effects model was used to calculate the pooled ORs and 95% CI if the *P*-value of heterogeneity tests was no more than 0.10 [36]; otherwise, a fixed-effect model was selected [37]. Further, we conducted subgroup tests stratified by ethnicity as well as genotyping method when more than one study was included. Sensitivity analyses were performed by excluding one study at a time and then calculating the pooled ORs by repeating the meta-analysis to assess the stability of the results. We used Begg’s funnel plot and Egger’s test to evaluate publication bias, with *P*<0.05 being considered significant publication bias [38].

## Results

### Characteristics of included studies

**Figure 1:**
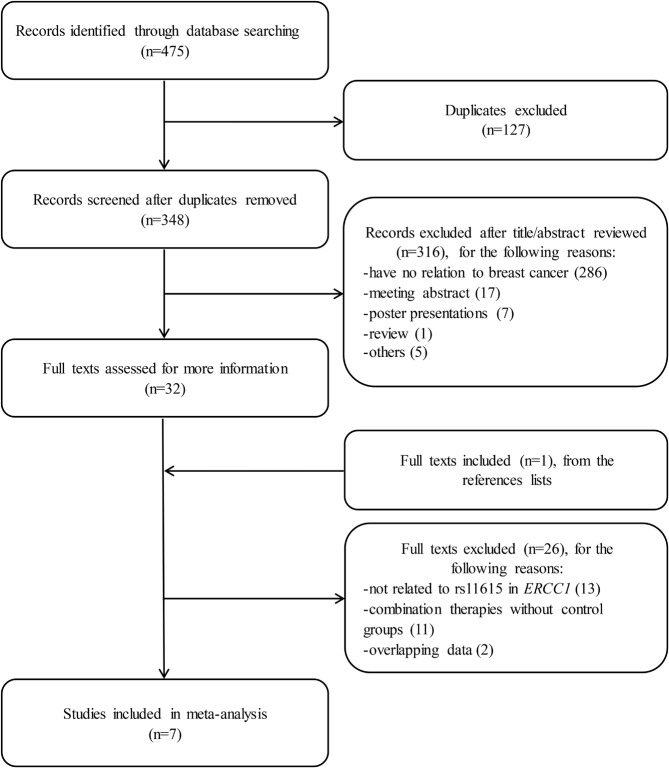
Flow diagram of article selection for our meta-analysis

A total of 475 publications were preliminarily identified after systematically searching the aforementioned databases based on our search strategy using different search term combinations. All articles were scrutinized carefully by reading the full texts, and the studies that conformed to the selection criteria stringently as mentioned above were selected for this meta-analysis. The detailed selection process is shown in Figure 1. In the end, seven case–control studies about the *ERCC1* rs11615 polymorphism and breast cancer risk were included in our study, encompassing 2354 cases and 2193 controls.

The characteristics of the eligible studies are shown in Table 1. The seven case–control papers were published between 2003 and 2017, and one study was performed in a Caucasian population, one in Mexican mixed population, and five in Asian population. All control subjects in these studies were within HWE, except for those conducted by Yang et al. [28] and Pongsavee et al. [32]. It came as a little surprise that there were two studies stratified by menopausal status (premenopausal and postmenopausal) with specific genotype data [27,31], and one committed to investigating postmenopausal breast cancer [26]. The information of premenopausal and postmenopausal subsets was also collected as we mentioned earlier (data not shown).

**Table 1:** Main characteristics of eligible studies in this meta-analysis

Author names	Years	Areas	Ethnicity	Genotyping method	Cases/controls	Cases			Controls			HWE	Association observed
						CC	CT	TT	CC	CT	TT		
Nexo et al. [26]	2003	Denmark	Caucasian	TaqMan	415/414	53	176	186	69	183	162	YES	No risk
Lee et al. [27]	2005	Korea	Asian	MALDI-TOF	705/550	411	257	37	323	187	40	YES	No risk
Yang et al. [28]	2013	China	Asian	TaqMan	461/504	183	166	112	232	184	88	NO	TT genotype showed increased risk
Zhu et al. [29]	2015	China	Asian	PCR-RFLP	101/101	56	41	4	63	33	5	YES	No risk
Gomez-Diaz et al. [30]	2015	Mexico	Mexican-mestizo	TaqMan	71/74	38	28	5	40	27	7	YES	No risk
He et al. [31]	2016	China	Asian	MALDI-TOF	450/430	230	195	25	261	151	18	YES	TT/TC genotype showed increased risk
Pongsavee et al. [32]	2017	Thailand	Asian	TaqMan	151/120	105	33	13	101	9	10	NO	TT/TC genotype showed increased risk

Abbreviation: PCR-RFLP, PCR-restriction fragment length polymorphism.

### Meta-analysis results

**Figure 2:**
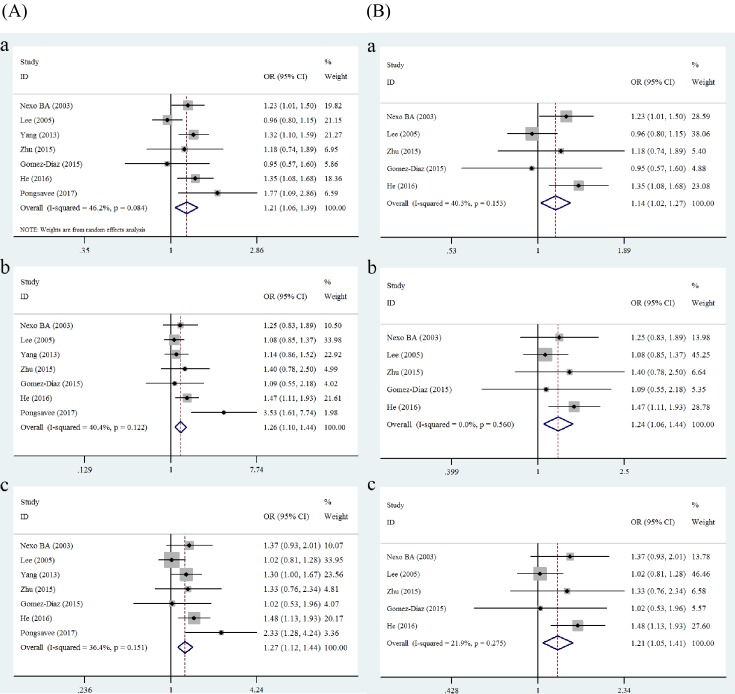
Forest plots of breast cancer risk associated with *ERCC1* rs11615 The plots were grouped into (**A**) and (**B**) for comparison. (A) All studies: (**a**) allele model (T compared with C); (**b**) heterozygous model (TC compared with CC); (**c**) dominant model (TC + TT compared with CC). (B) After excluding the studies that deviated from HWE: (**a**) allele model (T compared with C); (**b**) heterozygous model (TC compared with CC); (**c**) dominant model (TC + TT compared with CC).

The pooled results indicated that there is a statistically significant relationship between the *ERCC1* rs11615 polymorphism and increased breast cancer risk in all genetic models: the allele model (OR = 1.21, 95% CI = 1.06–1.39, *P*=0.006), homozygous model (OR = 1.29, 95% CI = 1.06–1.59, *P*=0.013), heterozygote model (OR = 1.26, 95% CI = 1.10–1.44, *P*=0.001), recessive model (OR = 1.20, 95% CI = 1.01–1.43, *P*=0.036), and dominant model (OR = 1.27, 95% CI = 1.12–1.44, *P*<0.001) (Table 2 and Figure 2). The next pooled analysis, which excluded studies that were not consistent with HWE, also suggested that *ERCC1* rs11615 was a breast cancer risk factor under the allele model (OR = 1.14, 95% CI = 1.02–1.27, *P*=0.02), heterozygote model (OR = 1.24, 95% CI = 1.06–1.44, *P*=0.007) and dominant model (OR = 1.21, 95% CI = 1.05–1.41, *P*=0.01) (Table 2 and Figure 2).

**Table 2:** Meta-analysis results of the association between the *ERCC1* rs11615 polymorphism and breast cancer susceptibility

Groups	Cases/controls	Genetic model	Test of association			Test of heterogeneity		Effects model	Begg’s test	Egger’s test
			OR	95% CI	*P* value	*I^2^*(%)	*P*_heterogeneity_		*P*	*P*
Overall	2354/2193	T compared with C	1.21	1.06–1.39	**0.006**	46.2	0.08	R	1.00	0.72
		TT compared with CC	1.29	1.06–1.59	**0.013**	35.2	0.16	F	0.13	0.38
		TC compared with CC	1.26	1.10–1.44	**0.001**	40.4	0.12	F	0.23	0.20
		TT compared with TC/CC	1.20	1.01–1.43	**0.036**	30.6	0.20	F	0.23	0.25
		TC/TT compared with CC	1.27	1.12–1.44	***P*<0.001**	36.4	0.15	F	0.76	0.35
Asians	1868/1705	T compared with C	1.24	1.03–1.50	**0.025**	61.3	0.04	R	0.81	0.49
		TT compared with CC	1.21	0.82–1.78	0.34	50.0	0.09	R	0.46	0.66
		TC compared with CC	1.35	1.05–1.74	**0.021**	59.6	0.04	R	0.22	0.13
		TT compared with TC/CC	1.10	0.76–1.60	0.60	48.8	0.10	R	0.46	0.47
		TC/TT compared with CC	1.33	1.07–1.66	**0.011**	54.9	0.06	R	0.22	0.22
HWE (*P*>0.05)*										
Overall	1742/1569	T compared with C	1.14	1.02–1.27	**0.02**	40.3	0.15	F	1.00	0.98
		TT compared with CC	1.14	0.87–1.48	0.34	40.5	0.15	F	0.81	0.71
		TC compared with CC	1.24	1.06–1.44	**0.007**	0	0.56	F	1.00	0.82
		TT compared with TC/CC	1.09	0.88–1.35	0.43	28.6	0.23	F	0.81	0.43
		TC/TT compared with CC	1.21	1.05–1.41	**0.01**	21.9	0.28	F	0.81	0.78
Asians	1256/1081	T compared with C	1.14	0.88–1.46	0.32	63.9	0.06	R	1.00	0.79
		TT compared with CC	0.96	0.67–1.37	0.81	46.2	0.16	F	1.00	0.82
		TC compared with CC	1.24	1.05–1.48	**0.013**	29.9	0.24	F	1.00	0.72
		TT compared with TC/CC	0.88	0.62–1.26	0.50	25.9	0.26	F	1.00	0.85
		TC/TT compared with CC	1.20	1.0–1.42	**0.03**	55.4	0.11	F	1.00	0.75

Abbreviations: F, fixed-effects model; *P* value, *P*-value for association; *P*_heterogeneity_, *P*-value for heterogeneity; R, random-effects model. P-values <0.05 are indicated in bold.

* The studies that clearly deviated from HWE were excluded.

### Subgroup analysis results

**Figure 3:**
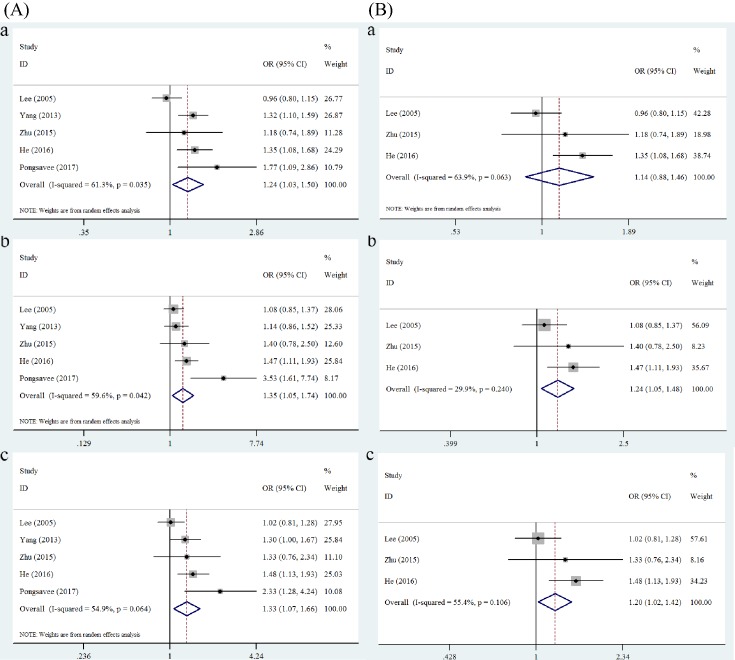
Forest plots of breast cancer risk associated with *ERCC1* rs11615 in Asian populations The plots were grouped into (**A**) and (**B**) for comparison. (A) All studies: (**a**) allele model (T compared with C); (**b**) heterozygous model (TC compared with CC); (**c**) dominant model (TC + TT compared with CC). (B) After excluding the studies that deviated from HWE: (**a**) allele model (T compared with C); (**b**) heterozygous model (TC compared with CC); (**c**) dominant model (TC + TT compared with CC).

Five case–control studies were included for subgroup analysis of Asian (China, Korea, Thailand) populations, while no subgroup analysis of the Caucasian population and Mexican-mestizo population was conducted, for each of which there was only study amongst the eligible studies. The stratified analysis showed an increased breast cancer risk in Asian populations based on the allele model (OR = 1.24, 95% CI = 1.03–1.50, *P*=0.025), heterozygote model (OR = 1.35, 95% CI = 1.05–1.74, *P*=0.021), and dominant model (OR = 1.33, 95% CI = 1.07–1.66, *P*=0.011) (Table 2 and Figure 3). We also found that there were two studies that deviated from HWE in this subset; further pooled results after excluding them indicated that the *ERCC1* rs11615 polymorphism still increased the breast cancer risk under the heterozygote model (OR = 1.24, 95% CI = 1.05–1.48, *P*=0.013) and dominant model (OR = 1.20, 95% CI = 1.02–1.42, *P*=0.03) (Table 2 and Figure 3).

Subgroup analysis of different genotyping methods (Taqman, MALDI-TOF, no PCR-restriction fragment length polymorphism (PCR-RFLP) for only one study) showed no relationship between *ERCC1* rs11615 and breast cancer risk, when including the studies that were deviant from HWE or not. In addition, when the data were stratified by menopausal status, there was no significant difference in the risk of breast cancer.

### Sensitivity analysis

**Figure 4:**
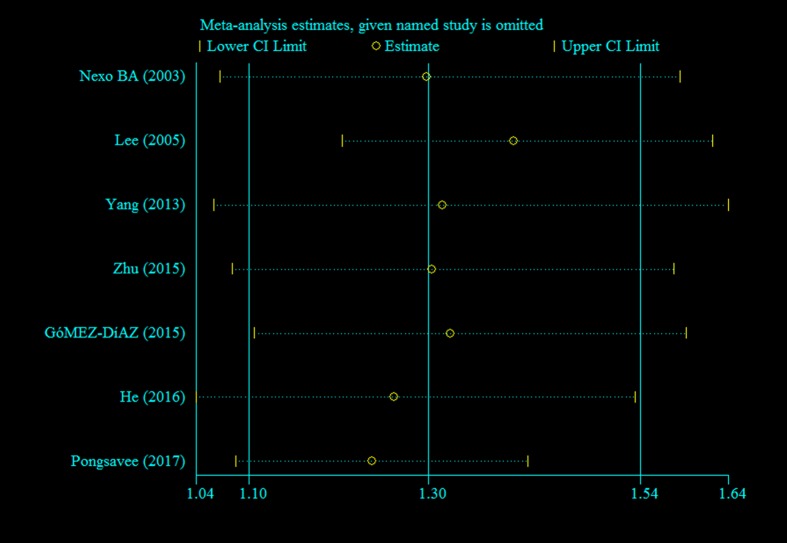
Sensitivity analysis of the *ERCC1* rs11615 polymorphism and breast cancer risk (dominant model: TC + TT compared with CC)

To observe the impact of each single study on the pooled OR, sensitivity analysis was performed by removing each study sequentially. In each case, the overall outcomes for the different genetic models showed no statistically significant changes, suggesting that this meta-analysis has good stability and reliability (Figure 4).

### Detection of heterogeneity and publication bias

**Figure 5:**
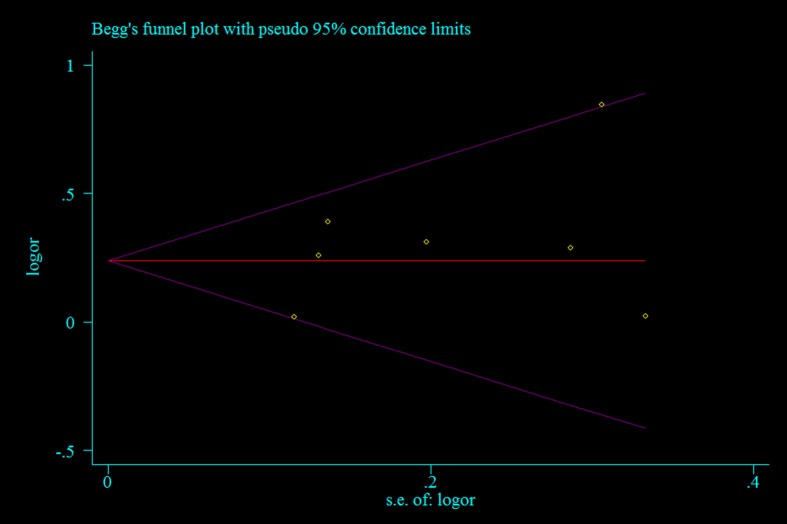
Funnel plot for evaluating publication bias in the seven studies (dominant model: TC + TT compared with CC)

Heterogeneity amongst the studies was evaluated using the *Q*-test and *I^2^* statistics. Substantial heterogeneity (*P*<0.10) was found, whereas no *I^2^*values were more than 75%. Thus, a model was applied to synthesize the data (Table 2). We use Begg’s funnel plot and the Egger’s test to evaluate publication bias. The funnel plot is symmetrical, indicating that there is no significant publication bias in the total population (Table 2 and Figure 5).

## Discussion

DNA repair systems play a vital role in maintaining the integrity and fidelity of the genome, and DNA repair capacity is a potentially important source of interindividual variability in relation to the development of cancer [39]. Particularly, polymorphisms in DNA repair genes can affect DNA repair capacity. Much attention has been drawn to heritable polymorphisms in DNA repair genes in relation to breast cancer risk; amongst such genes, *ERCC1* is highly polymorphic. Previous case–control studies focussed largely on the associations between breast cancer risk and two common variants, rs11615 and rs3212986. Although six prior studies have investigated the correlation between the *ERCC1* rs3212986 polymorphism and breast cancer development, no definite conclusions have been reached regarding this causal relationship [27,28,40–43]. The recent meta-analysis from Guo et al. [25] showed that amongst Caucasian populations, individuals with the rs3212986 polymorphism in the *ERCC1* gene have a higher risk of breast cancer. In the present meta-analysis, we paid special attention to the role of the rs11615 polymorphism in breast cancer risk. To date, there have been seven case–control studies devoted to shedding light on the link between the rs11615 polymorphism and breast cancer risk. The studies conducted by Yang et al. [28], He et al. [31], and Pongsavee et al. [32] found that the rs11615 polymorphism was associated with an increased risk of breast cancer, while others drew the opposite conclusion [26,27,29,30]. Here, we looked for powerful evidence that either supports or refutes the validity of associations between the *ERCC1* rs11615 polymorphism and breast cancer risk.

Our pooled study demonstrated a clear increase in breast cancer risk associated with the *ERCC1* rs11615 polymorphism under all genetic models (all *P*<0.05). We found no evidence of publication bias, and the results showed no instability through sensitivity analysis, convincing us of the reliability of the current meta-analysis. However, we took notice of deviations from HWE in the studies we included by Yang et al. [28] and Pongsavee et al. [32]. Genotyping errors, population stratification, and other genetic factors such as inbreeding or deletions can induce departure from HWE [44,45], which may, in some instances lead to false conclusions. To clarify if our meta-analysis results were altered by the studies by Yang et al. [28] and Pongsavee et al. [32], we ran an analysis again after excluding the data extracted from them. The increased risk of breast cancer under the allele model, heterozygous model and dominant model was still nominally significant (*P*=0.02, *P*=0.007, and *P*=0.01, respectively), which further confirmed the significant role played by *ERCC1* rs11615 in breast cancer susceptibility. The obvious alteration of *P*-values and estimates of effect size previously ascertained in all the genetic models could be due to reduced sample size, given that the two excluded studies accounted for 27.18% of individuals [46].

In further subgroup analysis by ethnicity, the *ERCC1* rs11615 polymorphism was associated with an increased risk of breast cancer in Asian populations. Unfortunately, there are no ample studies of Mexican-mestizo and Caucasian populations included in this meta-analysis. As a consequence, it is still too early to tell the role that the *ERCC1* rs11615 polymorphism plays in breast cancer risk variation in different ethnic backgrounds. Extremely large-scale single studies of different ethnicities are now necessary to draw a more precise conclusion about the specific significant association suggested by our intriguing finding. In addition, breast cancer is heterogeneous; De Waard et al. [47,48] showed a bimodal type of age distribution of breast cancer, which was considered to be related to menopause, and put forward the existence of two types of human breast cancer. In this meta-analysis, we tried to explore the role of the *ERCC1* rs11615 polymorphism in premenopausal and postmenopausal females. However, we did not find any difference between premenopausal and postmenopausal females in breast cancer susceptibility associated with *ERCC1* rs11615, contrary to the results by Lee et al. [27] and He et al. [31]. To date, the risk of breast cancer conferred by the *ERCC1* rs11615 polymorphism in relation to menopausal status has not been widely and thoroughly investigated and may be a promising area for breast cancer research.

Some limitations in the present meta-analysis should be noted. First, having only a total of seven papers, and even fewer after grouping, may limit the statistical power and result in some genuine associations being undetected. As previously described, it is unreasonable to state that there is a definite racial difference in the contribution of the *ERCC1* rs11615 polymorphism to breast cancer risk due to the insufficient number of studies. Second, the existence of heterogeneity in some models may generate false positive signals, while it could also lead to false negatives for some potential associations. We failed to discover any notable source of heterogeneity when we executed meta-regression analysis based on year, ethnicity, genotyping methods, and HWE in the control group (data not shown). Finally, compared with individual SNP loci, haplotypes may increase associations with disease. A study by Nexo et al. [26] found that by combining SNPs in the chromosomal region 19q13.2–3, they could detect a significantly increased risk of postmenopausal breast cancer, while none of the single SNPs showed this association [24]. The SNPs rs11615 and rs3212961 in *ERCC1* have strong or moderate linkage disequilibrium in Asian and European populations [49] and may be a significant haplotype. Our pooled results provide strong evidence of the increased risk of breast cancer for *ERCC1* rs11615, and Romanowicz et al. [9] concluded that Caucasian populations with the CC genotype of *ERCC1* rs3212961 have 10.61-times the risk of developing breast cancer. Thus, certain haplotypes may increase the strength of an individual polymorphism and lead to significant enrichment of essential biological functions. Unfortunately, no article in the present meta-analysis analyzed the frequency distributions of haplotypes except the one by Nexo et al. [26]. Further association analyses of *ERCC1* haplotypes are imperative.

In conclusion, our meta-analysis does demonstrate that the *ERCC1* rs11615 polymorphism significantly increases the risk of breast cancer. This significant association is observed in Asian populations, but more studies are necessary to determine if there is such an association in other races. Taking all our findings together, the increased risk of breast cancer under the heterozygote model and dominant model persisted, revealing that the TC genotype plays a crucial role in determining breast cancer risk. Well-designed case–control studies with larger sample sizes and examining populations from across the world are essential for the exploration of the association between breast cancer risk and *ERCC1* gene polymorphisms, other DNA repair gene polymorphisms, and even the relevant haplotypes.

## Abbreviations

CI: confidence interval
ERCC1: excision repair cross-complementation group 1
HWE: Hardy–Weinberg equilibrium
OR: odds ratio
SNP: single-nucleotide polymorphism
XPF: xeroderma pigmentosum complementation group F
